# Understanding of and reasoning about object–object relationships in long-tailed macaques?

**DOI:** 10.1007/s10071-012-0591-x

**Published:** 2013-02-17

**Authors:** Christian Schloegl, Michael R. Waldmann, Julia Fischer

**Affiliations:** 1 Cognitive Ethology Laboratory, German Primate Center Göttingen, Kellnerweg 4, 37077 Göttingen, Germany; 2 Courant Research Centre “Evolution of Social Behaviour”, Georg-August-University Göttingen, Kellnerweg 6, 37077 Göttingen, Germany; 3 Department of Psychology, Georg-August-University Göttingen, Goßlerstrasse 14, 37073 Göttingen, Germany

**Keywords:** Long-tailed macaque, Diagnostic reasoning, Inference, Monkey, Causality, Object knowledge

## Abstract

Diagnostic reasoning, defined as the ability to infer unobserved causes based on the observation of their effects, is a central cognitive competency of humans. Yet, little is known about diagnostic reasoning in non-human primates, and what we know is largely restricted to the Great Apes. To track the evolutionary history of these skills within primates, we investigated long-tailed macaques’ understanding of the significance of inclinations of covers of hidden food as diagnostic indicators for the presence of an object located underneath. Subjects were confronted with choices between different objects that might cover food items. Based on their physical characteristics, the shape and orientation of the covers did or did not reveal the location of a hidden reward. For instance, hiding the reward under a solid board led to its inclination, whereas a hollow cup remained unaltered. Thus, the type of cover and the occurrence or absence of a change in their appearance could potentially be used to reason diagnostically about the location of the reward. In several experiments, the macaques were confronted with a varying number of covers and their performance was dependent on the level of complexity and on the type of change of the covers’ orientation. The macaques could use a board’s inclination to detect the reward, but failed to do so if the lack of inclination was indicative of an alternative hiding place. We suggest that the monkeys’ performance is based on a rudimentary understanding of causality, but find no good evidence for sophisticated diagnostic reasoning in this particular domain.

## Introduction

On my way to the office on a stormy autumn day, I observe the trees bending and the leaves whirling around. Naturally and without giving it much thought, I am aware that it is the wind that causes the movement of the trees and not the trees that cause the wind. In other words, I understand the causal relationship and can use this knowledge to infer the effect of wind on a treeless sandy beach. We think about such causal relationships regularly in our everyday life; it is an important mechanism allowing us to make sense of our world (Waldmann and Hagmayer 2013). However, if and to what degree non-human animals also reason about causes and effects is not clear and the centre of some fierce debates (see, for example, Blaisdell and Waldmann 2012; Penn and Povinelli 2007; Waldmann et al. 2012). While the precise nature of causal reasoning remains disputed, there is increasing evidence that at least primates and corvids may understand the causally relevant features of objects (e.g., Hanus and Call 2011; O’Connell and Dunbar 2005; Taylor et al. 2011; Visalberghi et al. 2009) and may learn discriminations based on causal cues faster than those based on arbitrary factors (Hanus and Call 2011).

Understanding cause–effect relationships lays the groundwork from which one can reason about one factor if observing the other factor. Importantly, such causal reasoning works in two directions: one can identify the effect of a given cause (predictive reasoning), but also the cause of an observed effect (diagnostic reasoning). This distinction between predictive and diagnostic reasoning has been investigated in a number of studies in human psychology (Fernbach et al. 2011; Waldmann 2000) but not in the animal cognition literature. Here, both directions are usually subsumed under “causal reasoning”.

The first reports about diagnostic reasoning (according to our definition) in non-humans comes from work by Premack and Premack (1994). In one of their experiments, chimpanzees (*Pan troglodytes*) saw an experimenter hiding two different types of food under two containers. Then, an experimenter first removed, unseen by the chimpanzees, one of the two fruits before eating it in plain sight of the animals. Subsequently, the subjects were allowed to approach the containers. One of four chimpanzees apparently detected the underlying causation that the experimenter had eaten, say, the apple, because he had removed it from the container; as a consequence, this chimpanzee then approached the other container to obtain the remaining fruit (see also Call 2006 and Mikolasch et al. 2011 for similar results with chimpanzees and Grey parrots *Psittacus erithacus*). One of the difficulties for the subjects in these studies is that they have to infer first that the presentation of the food means that this food has been removed from its hiding place; in a second step, they have to infer that they have to choose the alternative container to obtain the other reward. Similar two-step inferences are involved in the “shaking” task. Here, apes and Grey parrots seem to understand that (a) rattling noises emitted during the shaking of a bowl are indicative for the presence of a reward in this bowl (first inference) and that (b) the absence of noise is indicative for the absence of a reward, and therefore, the other bowl must be chosen (second inference; e.g., Call 2004; Schloegl et al. 2012). Capuchin monkeys (*Cebus apella*) do not succeed instantaneously in this task, but may come to understand this relationship through experience (e.g., Heimbauer et al. 2012; Sabbatini and Visalberghi 2008; see also Maille and Roeder 2012 for tentative evidence in lemurs).

Using a different approach, Call (2007) was interested in Great Apes’ knowledge about the effect objects can have on the spatial orientation of other objects. To illustrate this, imagine an apple hidden under a cardboard. Because of the influence of the apple on the orientation of the board, it would not be lying flat on the ground but would be inclined. Thus, understanding that the inclination is caused by the apple would allow an observer to reason diagnostically about the location of the apple. Similarly, the absence of an inclination would be indicative of the presence of the apple in a different location. In his study, Call confronted his subjects first with a food reward hidden underneath one of two wooden boards, which was inclined. The results demonstrated that apes use the inclination of the board as an indicator for the presence of the reward underneath the board. Notably, in a later task, the apes first saw that a large piece of a less preferred food item and a smaller piece of a more preferred food item were to be hidden; after the hiding, however, the subjects chose against their food preference and selected the board with the stronger inclination. This preference was particularly pronounced in bonobos (*Pan paniscus*) and gorillas (*Gorilla gorilla*), but was less obvious in orang utans (*Pongo* sp.). Taken together, the apes were able to use the observation of an effect (presence of an inclination) to detect its cause (presence of the food), but their abilities were limited: They could not use the strength of the inclination to determine which food had been hidden where, but preferred the strongest inclination instead (Call 2007). Interestingly, as in the apes, human children’s physical knowledge analogously develops gradually and context specifically (e.g., Baillargeon 1991, 2004).

To assess the evolution of human cognition, it is important to trace the origin of a variety of cognitive abilities through the primate lineage (MacLean et al. 2012). In recent years, several approaches have been taken to assess the cognitive abilities of a variety of species for cross-species comparisons (e.g., Amici et al. 2008, 2010; Herrmann et al. 2007; Hill et al. 2011). One interesting finding is that monkeys performed more similar to the Great Apes than expected in a wide set of tasks (e.g., Amici et al. 2010; Herrmann et al. 2007; Schmitt et al. 2012). Thus, the differences in brain size between apes and monkeys may not translate into cognitive differences in all tasks but in superior performances of apes in some selected cognitive tasks only (e.g., mirror self-recognition or perspective taking; Anderson and Gallup 2011; Hare et al. 2000, 2003). In a recent study applying the primate cognition test battery (PCTB as developed by Herrmann et al. 2007), long-tailed macaques *Macaca fascicularis* showed some indication for diagnostic understanding about the effects objects can have on other objects: When the monkeys had to choose between an inclined board and a board lying flat on the ground, with food hidden under the inclined board, they performed similarly to the Great Apes by choosing the board with the reward underneath (Schmitt et al. 2012). One drawback of such large-scale comparisons using a wide variety of tasks is, however, that they rarely tap into the cognitive mechanisms by which the subjects’ performances are achieved. For instance, Schmitt et al. (2012) conducted six trials only and did not include control conditions. Thus, from their results, we can conclude that monkeys indeed prefer inclined boards; however, it did not become clear if the monkeys chose the inclined board because it was perceptually more salient than the flat board, or if they understood that something had *caused* the inclination. And if the monkeys did understand the underlying causality, would they also be able to use this knowledge to reason diagnostically?

To answer these questions, we designed a new set of tasks for which we made use of the monkeys’ previous experiences with inclined boards. Thus, we were not interested if the monkeys would choose inclined boards (as this was already known), but we were interested in exploring in-depth the cognitive mechanisms and the monkeys’ understanding of causality. To get information about the cognitive mechanisms, we employed a series of tasks of decreasing complexity (see Seed et al. 2012, for a similar approach). In the initial study, we presented two of three potentially available objects on each trial and each object would be differently influenced by a food reward placed underneath (e.g., a cup, a board and a board resting on a wooden block). Thus, this task required the subjects to track three different objects and base their decision where to expect the reward on the type of objects and their initial and final orientation. In the following studies, we sequentially removed potentially confounding or distracting factors from this basic design to titrate the boundary conditions of long-tailed macaques’ understanding of causality and diagnostic reasoning. We predicted that if the salience of the inclination explains the monkeys’ choices, they should prefer inclined boards regardless of whether or not the inclination is indicative of the reward. If they understand the causality, however, they should choose the inclined board only if the orientation had changed due to the hiding of a reward. Likewise, they should not prefer a solid object with an inclined surface, as this inclination is an inherent feature of the object but not diagnostic for a reward (see also Call 2007). Finally, if the underlying choice mechanism is based on fully developed diagnostic reasoning, the macaques should apply this skill flexibly and generalise it to other cause–effect relationships, for instance if a reward is hidden under a hollow cup instead of a board.

## Experiment 1: differential influences of a reward on three different objects

### Methods

#### Subjects and housing

We tested seven long-tailed macaques (three males), which were housed in a group of 31 individuals at the German Primate Center, Göttingen. At the time of testing, the subjects were between 2 and 9 years old (mean age: 4.1 years; Table 1). The only criterion to include subjects in this and the subsequent experiments was their willingness to participate in the tasks. Housing facilities consisted of indoor (25 m²) and outdoor areas (141 m²), and subjects were tested individually in a small cage (42 × 58 × 57 cm (*b* × *l* × *h*) connected to a testing compartment (approx. 1 m², 2.3 m high) inside their familiar indoor area. The subjects were free to leave the cage and enter the testing compartment at any time during testing. Tests were conducted once per day and participation was voluntary, that is, dependent on the monkeys’ willingness to enter the test compartment and the cage. Water was available ad libitum, and monkeys were not food deprived. All subjects had participated in the “Shape” task of the PCTB (Schmitt et al. 2012).

**Table 1:** Subjects and participation in the experiments

Subject	Age (years)	Sex	Participation in experiment			
			1	2	3	4
Ismael	3	♂	✔	✔	✔	
Maja	4	♀	✔	✔	✔	✔
Paule	4	♂	✔			
Popeye	4	♂	✔	✔		
Selina	3	♀	✔	✔	✔	✔
Sophie	2	♀	✔	✔	✔	✔
Sunny	9	♀	✔	✔	✔	✔
Lennie	2	♂		✔	✔	✔
Samson	3	♂		✔	✔	✔
Sally	4	♀			✔	✔

#### General procedure

The monkeys saw the setup through a transparent Plexiglas pane attached to the front side of the cage. Objects were presented on a sliding platform made of grey polyvinylchloride (80 × 27 cm) affixed to the Plexiglas pane. Three small holes (diameter: 1 cm; distance between the holes: 15.5 cm) were drilled into the pane, and objects were positioned in front of the left and right hole. During the presentation of the setup, the platform was pushed backward to a distance of approx. 6 cm between the pane and the front edge of the platform. After the presentation, the platform was pushed forward towards the pane and the subjects made a choice by sticking a finger through the hole in front of an object. In a few cases, the monkeys’ choices were not unequivocal, as they put a finger in each of the two holes or switched rapidly between the two holes. In these cases, the experimenter pulled back the platform, waited a few seconds and pushed the platform forward again. The experimenter sat opposite of the monkey in reaching distance of the platform.

#### Choice training

Even though the subjects had participated in choice tasks before, we conducted training sessions to ensure that the monkeys were accustomed to choosing between one of two objects. The experimenter placed a food reward (pieces of fresh banana, grape, apple, pear or plum or pieces of dried plum or apricot, depending on availability and individual preferences) on the platform in front of one of the two outer holes; he then simultaneously positioned two identically looking white cups (10 cm in height and 6.5 cm in diameter) on the platform, with one of the two cups covering the food. The other cup was placed in front of the other outer hole. The platform was pushed towards the monkey, and the animal could indicate its choice. The experimenter remained seated behind the platform with the hands resting in his lap and the face looking straight ahead, neither looking directly at the setup nor the monkey. If the correct cup was chosen, the experimenter handed the food to the animal. If not, the reward was removed from the cup, shown to the monkey and discarded. The food was hidden randomly on the left or on the right side, with the restriction that the food was not on the same side for more than three consecutive trials. A session lasted for 12 trials and because the monkeys were familiar with choice procedures, we advanced them to testing if they chose the baited cup on at least eleven of 12 trials of a single session (*P* = 0.006 in a two-tailed binomial test).

#### Testing

In the actual tests, a food reward was hidden underneath one of two different objects behind an opaque screen (50 × 20 cm). In total, three different types of covering objects were available, but only two were presented on each trial. As objects we used (a) a plastic cup (5 cm in diameter and 4 cm in height) laminated with brown tape, (b) a rectangular piece of cardboard (13 × 10 cm), also laminated with brown tape and (c) an identical piece of cardboard, resting on a square wooden block (edge length: 2 cm); the monkeys saw that the wooden block caused the inclination of the cardboard. In the following, we will refer to these three objects as “cup”, “flat board” and “pre-inclined board”, describing their appearance prior to the hiding of the reward.

At the start of each trial, two of these objects were put on the platform in full view of the subjects, with the position of the objects (left/right) randomised. The experimenter lifted the objects repeatedly to demonstrate that no food was present; thereby, the subjects could clearly see that the inclined board was resting on the wooden block only. Next, the experimenter put the screen between the subjects and the setup, took a piece of food out of a food bowl and lifted his hand above the screen to show the reward to the subject. He then lowered his hand behind the screen and manipulated first the object on the left side and then the object on the right side. Manipulations either consisted of hiding the food underneath the object or only touching and re-arranging it. Finally, the experimenter lifted his hand again, opened his palm to demonstrate the food is no longer in his hand, removed the screen and pushed the platform towards the subjects, which were then allowed to make a choice. The experimenter lifted the selected object and, if the subjects had made a correct choice, he handed the reward to the monkey. Otherwise, he lifted the other object to reveal the location of the reward and removed it. The objects were then re-arranged and the next trial started. The location of the reward was randomised with the stipulation that the same object or the same side was not rewarded for more than three consecutive trials.

**Fig. 1:**
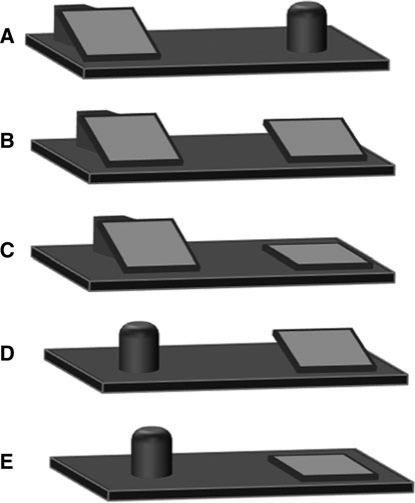
Illustrations of the five different conditions presented in Experiment 1, shown from the perspective of the monkeys. The objects are shown as orientated after the hiding of the reward. In Condition *A*, the reward was under each of the two objects in 50 % of the trials. In Condition *B*, the reward was under the board that had been flat before the hiding (the board on the right side in this example). In Condition *C*, the reward was under the inclined board that rests on a wooden block. In condition *D*, the reward was under the board and in Condition *E*, it was under the cup

In total, we administered five different conditions (see Fig. 1):

*Condition A (control)*: The pre-inclined board and the cup were presented. In half of the trials, the food was hidden underneath the board, in the other half of the trials underneath the cup. Because hiding of the food did not change the orientation of any of the two objects, subjects were not expected to be able to detect the location of the food.

*Condition B*: The pre-inclined board and the flat board were presented, and the food was hidden underneath the flat board; thus, both boards were inclined after the hiding of the food.

*Condition C*: As condition B, but the food was hidden underneath the pre-inclined board; thus, the flat board remained lying flat on the platform.

*Condition D*: The flat board and the cup were presented, and the food was hidden underneath the flat board; thus, the flat board was inclined after the hiding of the food, whereas the cup remained unchanged.

*Condition E*: As in Condition D, but the food was hidden underneath the cup; thus, the flat board remained lying flat on the platform.

We were interested whether the monkeys could use the change of orientation of the flat board or the absence thereof to infer the location of the food. Furthermore, we wanted to assess if the monkeys could differentiate between causally relevant and irrelevant changes of board orientation. Therefore, in half of the trials of conditions B–E, we additionally rotated the flat board by 90°. Thus, we created trials in which

(a): no modifications occurred (no rotation of the flat board in conditions C and E)
(b): causally irrelevant modifications occurred (rotation of the flat board in conditions C and E)
(c): causally relevant changes occurred (inclination, but no rotation of the board in conditions B and D) and
(d): causally relevant and irrelevant changes occurred (inclination and rotation of the flat board in conditions B and D).

The same types of reward were used as during the training sessions. Note that the pieces of food were smaller than the block underneath the pre-inclined board (approximate size: 1 cm, with some small variation due to the characteristics of the fruits); this was done to ensure that hiding an additional piece of food underneath the pre-inclined board would not alter the inclination of the board; however, as a consequence, the inclination of the flat board was less steep than the inclination of the pre-inclined board.

A session lasted for a maximum of 12 trials and if subjects terminated a session pre-maturely, the missing trials were run on the next day, up to a maximum of 12 trials per session. For analysis, we formed blocks of 12 consecutive trials each. Subjects received 8 blocks of 12 trials for a total of 96 trials; within a block, conditions B–E were presented twice each, with four trials of condition A interspersed. This was done to ensure that food was hidden an equal amount of times under each object and that the monkeys would not form preferences for certain objects because of an increased likelihood of finding food underneath a specific object. The order of conditions was randomised. Because of an experimenter error, a few sessions were terminated after 10 trials. Missing trials were conducted in a different session. Subjects received only 1 session/day.

#### Analysis

All trials were videotaped for later analysis. A second rater coded eleven sessions (approx. 20 % of all trials) and the inter-observer reliability was excellent (98.5 % agreement, Cohen’s *Κ* = 0.967). For the analysis, we scored the percentage of correct choices in each condition. We further measured if the subjects had side biases, that is, a preference for the objects to their left or right side.

Most data were normally distributed and consequently we used parametric statistics whenever appropriate. Repeated measures ANOVAS with the Holm–Sidak post hoc procedure were applied to compare performances between conditions, and one-sample *t* tests to assess if group performances within each condition deviated from chance. Only in one case, the assumption of normality was not fulfilled and therefore we used a Wilcoxon test instead. Binomial tests were used to analyse individual performances. We used Pearson correlations or Spearman correlations (depending on distribution of the data) to test for performance changes across blocks. We present exact, two-tailed p-values throughout, with α = 0.05. Tests were conducted using SPSS 11.5 and SigmaPlot 11.

### Results

#### Training

Six of the seven monkeys reached the training criterion in the first session, with five of these monkeys not making any mistake. One male reached the criterion after three sessions.

#### Test

We first assessed whether the monkeys’ performance was influenced by the causally irrelevant modification (rotation of the flat board by 90°) in half of the trials in the conditions B–E. Therefore, we ran a two-way repeated measures ANOVA and included “with or without rotation” as a factor. We also included “condition” (B–E), to assess if an effect of rotation may have been dependent on the condition. As the flat board was not used in the control condition A, it was not included in the analysis. We found performance differences between the conditions (*F*_3,18_ = 3.376, *P* = 0.041), but neither an effect of the 90° rotation of the flat board (*F*_1,18_ = 0.067, *P* = 0.805) nor an interaction between the factors (*F*_3,18_ = 0.488, *P* = 0.695). Additionally, we ran a one-way repeated measures ANOVA to assess if the performance differed between trials in which no modification, only causally relevant, only causally irrelevant or both types of modifications occurred. This was not the case (*F*_6,18_ = 0.209, *P* = 0.889). Thus, the causally irrelevant rotation of the flat board had apparently no influence on the monkeys’ performance, and we subsequently pooled within each condition the trials with and without the 90° rotation of the flat board.

**Fig. 2:**
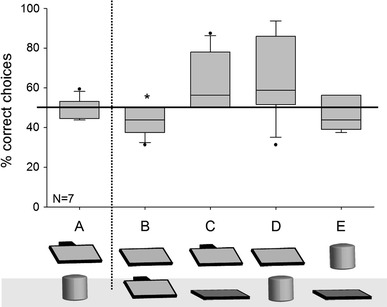
Performance of the monkeys in Experiment 1. *Capital letters* *below* the *x* axis denote the conditions. The two objects used per condition are shown below the *x* axis. The illustration shows the objects after the hiding of the reward. For conditions *B*–*E*, the *top* object is rewarded and the *lower* object is not rewarded. In the control condition *A*, each object was rewarded on 50 % of the trials. *Boxplots* show median, 25th and 75th quartile, *whiskers* show 10th and 90th percentiles, *dots* represent outliers. The *solid horizontal line* represents the 50 % chance level, the *vertical dotted line* separates control and test conditions. *Asterisk* shows significant deviation from chance according to a one-sample *t* test

Next, we conducted a one-way repeated measures ANOVA to assess performance differences between all five conditions. Although we found significant differences (*F*_4,24_ = 3.23, *P* = 0.03) in the global test, the post hoc tests all turned out to be non-significant (all *P*s ≥ 0.101; Fig. 2). Contrasting the monkeys’ performance in each condition with the theoretical chance level of 50 % revealed chance performance in the control condition A (one-sample *t* test: *t* = 0, *df* = 6, *P* > 0.999) as well as in the conditions D (flat board and cup, with food hidden underneath the flat board: *t* = 1.595, *df* = 6, *P* = 0.162) and E (flat board and cup, with food hidden in the cup: Wilcoxon test: *T* ^+^=19, *df* = 6, *P* = 0.469). In condition B (pre-inclined and flat board, food hidden underneath the flat board) the monkeys had a small, yet significant preference for the un-baited, pre-inclined board (*t* = −2.489, *df* = 6, *P* = 0.047), whereas in Condition C (pre-inclined and flat board, food hidden underneath the pre-inclined board) the same preference for the pre-inclined board marginally failed to reach significance (*t* = 2.228, *df* = 6, *P* = 0.067; Fig. 2).

Visual inspection of Fig. 2 suggests at least a marginally better performance in conditions C and D, that is, in those conditions in which only one board was inclined under which the food was hidden, than in the other conditions. This effect, however, is driven by the superior performance of two females who both chose the rewarded object significantly above chance in both conditions (binomial tests, all *P*s ≤ 0.021), whereas all other performances by these and the other monkeys were at chance level (all *P*s ≥ 0.21).

No clear preference for one of the three objects was detectable (one-way repeated measures ANOVA: *F*_2,14_ = 2.796, *P* = 0.095), but strong side biases occurred in six of the seven subjects (binomial tests, all *Ps* ≤ 0.032). Interestingly, we found a decrease in choice accuracy in later blocks (Spearman: *N* = 8, *r*_*s*_ = −0.916, *P* < 0.001). The oldest subject, which was more than twice as old as all other monkeys (Table 1), did not perform differently than the other subjects.

### Discussion

This first experiment showed that the long-tailed macaques had considerable problems to detect the location of the hidden food based on the spatial orientation of the objects under which the reward could have been hidden. In contrast to this, the same subjects were successful in a significantly simpler task in which the reward was hidden underneath one of two boards, which had both been lying flat on the ground prior to the hiding of the reward (Schmitt et al. 2012). Whereas the superior performance of the subjects in that task could have been based on a preference for an inclined board only, they here had to make several considerations to be successful: they had to take into account (1) if an object’s spatial orientation would be influenced by a reward hidden underneath (board vs. cup), (2) if a board had been inclined prior to the hiding, and (3) if this pre-inclined board could still be the location of the reward; this last consideration was furthermore dependent on the type and orientation of the other object. In other words, the computational demands of this task may have been too high. A similar argument had been raised by Call (2007); (see also Seed et al. 2012), whose apes failed to distinguish between two inclined boards, one of which was resting visibly on a wooden block. This possibility is supported by the success of two of our female subjects in the conditions in which they could base their choice on the presence of a single inclination only. Thus, in principle, some long-tailed macaques are able to rely on a board’s inclination to detect the food underneath; however, the majority of the monkeys failed to do so. Beside the computational demands of the task, there are several sources of distraction that may have influenced the other subjects. First, to assess if the monkeys could distinguish between diagnostically relevant and irrelevant modifications, we included the irrelevant 90° rotation of the flat board in half of the trials. This may have distracted the monkeys, even though the lack of an effect of the rotation renders this possibility unlikely. Second, the strength of the two inclinations differed, which may have biased the monkeys towards the pre-inclined board and may have made it more difficult to distinguish between the pre-inclined and the flat board. There is tentative support for this assumption: Even though we did not find an overall preference for the pre-inclined board, the monkeys’ performance was worst in the condition in which both boards had been inclined; only in this condition the subjects performed below chance, indicating a weak but detectable bias towards the stronger inclination. Thus, the long-tailed macaques may be subjected to the same bias as bonobos and gorillas (but not orang utans; Call 2007). Finally, most monkeys showed severe side biases, a common phenomenon in unsuccessful subjects and may be seen as a fallback strategy in over-demanding tasks.

## Experiment 2: reducing the computational demands—flat board versus cup

In this experiment, we simplified the task to test if the computational demands of the first experiment may have masked the cognitive abilities of the long-tailed macaques. Therefore, we first excluded the 90° rotation of the flat board, as this manipulation had been ignored by the monkeys. Second, we removed the pre-inclined board, as the occurrence of two inclined boards may have exceedingly increased the task’s complexity. Yet, we continued to confront the monkeys with the flat board and the cup. Thus, to solve the tasks, the monkeys would still need to consider the dependence of the influence of the reward on the objects’ properties.

### Methods

#### Subjects

Six of the seven subjects (two males) from Experiment 1 participated, while the third male was not motivated to enter the test compartment. In addition, two other males (2 and 3 years old) participated (Table 1). Both new subjects had participated in the study by Schmitt et al. (2012).

#### General procedure

The general procedure was identical to Experiment 1.

#### Training

We used a transposition task (e.g., Rooijakkers et al. 2009) for training. In this task, the monkeys had to track the movement of a baited cup while simultaneously tracking the movement of a distractor cup. The idea was to prime the monkeys to pay attention to the setup and thereby counteracting their side biases. The monkeys could observe how one piece of food was hidden underneath one of two identical white plastic cups (10 cm in height and 6.5 cm in diameter). Then, the experimenter simultaneously touched each cup with one hand and executed one of two different manipulations:

*Transposition*: the two cups were moved slowly and simultaneously from their original side of the platform to the opposite side of the platform, that is, from the left side to the right side or vice versa. Thereby, each cup replaced the other cup in its original position. During the movement, the cups were crossing their paths in the middle of the platform.

*No transposition*: both cups were moved slowly and simultaneously to the middle of the platform, but were then returned to their original position; thus, no crossing of the paths occurred.

To ensure that the monkeys watched the entire procedure, the manipulations were conducted slowly and the speed was adjusted to the monkeys’ attention: if a monkey shifted its attention and looked away from the setup, the movement was paused and the experimenter called the monkey’s name. The movement was resumed as soon as the monkey looked at the setup again. The location of the food and the order of manipulation were randomised with the stipulation that neither side was baited for more than three consecutive trials; similarly, the same manipulation was not repeated for more than three consecutive trials.

Each session consisted of 12 trials, with each manipulation presented six times. If subjects terminated a session pre-maturely, the missing trials were conducted on the next day, up to a maximum of 12 trials per session. For the analysis, we formed blocks of 12 consecutive trials. To advance to testing, the monkeys had to choose the baited cup on at least 10 of the 12 trials for two consecutive blocks and with no more than one error per manipulation per block (for each session *P* = 0.039 in a two-tailed binomial test).

#### Testing

The procedure was identical to Experiment 1, but to make it salient that the task differed from the previous one, we used new cups and boards. Cups measured 6 cm in diameter and 6 cm in height, boards measured 13 × 13 cm. All objects were covered by black tape.

We administered four different conditions:

*Condition A (control)*: two cups were presented, and the reward was hidden randomly underneath one of them. Because hiding of the food did not change the orientation of any of the two objects, subjects were not expected to be able to detect the location of the food.

*Condition B*: two flat boards were presented; after the hiding of the reward underneath one of the boards it was inclined.

*Condition C*: a flat board and a cup were presented; the reward was hidden underneath the board causing an inclination of the board.

*Condition D*: as in condition C, but the reward was hidden underneath the cup so that the board remained lying flat on the platform.

A session lasted 12 trials and subjects received 6 blocks of 12 trials for a total of 72 trials; within a session, each condition was presented three times, for a total of 18 trials per condition. The order of conditions was randomised. Subjects received only 1 session/day.

#### Analysis

Data were analysed in the same way as in Experiment 1.

### Results

#### Training

The monkeys needed 6.4 ± 4.1 sessions (*x* ± SD; range 2–14) to reach the training criterion. More errors occurred in the “transposition” than in the “no transposition” condition (71.9 ± 21.3 % of all errors; *x* ± SD; paired *t* test: *t* = 2.905, *df* = 7, *P* = 0.023).

#### Test

**Fig. 3:**
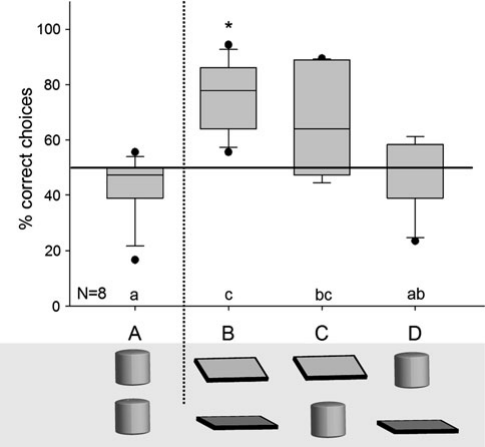
Performance of the monkeys in Experiment 2. *Capital letters* below the *x* axis denote the conditions. The objects used per condition are shown below the *x* axis. The illustration shows the objects after the hiding of the reward. For conditions *B*–*D*, the *top* object is rewarded and the *lower* object is not rewarded. In the control condition *A*, each object was rewarded in 50 % of the trials. *Boxplots* show median, 25th and 75th quartile, *whiskers* show 10th and 90th percentiles, *dots* represent outliers. The *solid horizontal line* represents the 50 % chance level, the *vertical dotted line* separates control and test conditions. *Boxes* marked with *small letters* *above* the *x* axis differ significantly from each other, based on a repeated measure ANOVA with post hoc Holm–Sidak tests. *Asterisk* shows significant deviation from chance according to a one-sample *t* test

A repeated measures ANOVA revealed significant performance differences between the conditions (*F*_3,21_ = 7.143, *P* = 0.002), and post hoc analyses show that the subjects were significantly more successful in conditions B and C, in which the reward was hidden underneath an inclined board, than in the control condition A (*Ps* ≤ 0.035; Fig. 3); furthermore, subjects were more successful in condition B (two boards) than in condition D (one board and one cup, with the food underneath the cup; *P* = 0.012). The contrast between conditions C and D (one board and one cup; in C the reward is underneath the board, in D it is underneath the cup) failed to reach significance (*P* = 0.083), whereas all other comparisons were non-significant (all *P*s ≥ 0.494). Similarly, when contrasting the performance within each condition with the hypothetical chance level of 50 %, the monkeys were significantly above chance in condition B (one-sample *t* test: *t* = 5.225, *df* = 7, *P* = 0.001), whereas they failed to reach the significance threshold in condition C (Wilcoxon: *T* ^+^ = 4, *df* = 7, *P* = 0.109); the monkeys’ choices were at chance level in conditions A (*t* = −1.57, *df* = 7, *P* = 0.16) and D (*t* = −0.52, *df* = 7, *P* = 0.619; Fig. 3). Four and three monkeys were significantly above chance in conditions B and C, respectively (binomial test, all *P*s ≤ 0.031), but none was in conditions A and D. The two subjects that had not participated in Experiment 1 did not perform differently than the other monkeys: One of the subjects was among the best-performing individuals and solved condition B significantly above chance but marginally failed to pass the significance threshold in condition C (13 of 18 trials correct; *P* = 0.096), while the other subject chose at chance level in all conditions. Again, the oldest subject performed at the same level as the other monkeys. The performance of the monkeys did not change over the course of the six blocks (all conditions combined: Pearson: *r* = −0.204, *P* = 0.698). Likewise, in none of the conditions, a change was observed when comparing the performance in the first three blocks and the performance in the second three blocks (paired *t* test or Wilcoxon, as appropriate: all *P*s ≥ 0.438). Finally, five of the eight monkeys again revealed significant side biases (binomial tests, all *P*s ≤ 0.024).

### Discussion

In this second experiment, the monkeys’ performance increased relative to the first experiment; a significant number of individual subjects was able to use the occurrence of an inclination of the board to detect the reward. It is noteworthy that the choice accuracy of the two subjects who had not participated in Experiment 1 did not deviate from the accuracy of the other monkeys, suggesting that the improved performance is not attributable to learning across experiments. Rather the reduction in complexity allowed the monkeys to detect the regularities of the task and to be successful. Under these test conditions we were able to replicate the original finding of Schmitt et al. (2012), suggesting that long-tailed macaques are able to use the presence of an inclination to find a reward hidden underneath this board. Furthermore, the long-tailed macaques seem to match the performance of the Great Apes, which had been tested repeatedly in a task in which food was hidden under one of two boards (similar to condition B; Bräuer et al. 2006; Call 2007; Herrmann et al. 2007). However, our results also suggest that the long-tailed macaques’ abilities may not go much beyond this level of complexity and may not be based on elaborate diagnostic reasoning, as the monkeys were still unable to use the absence of an inclination to infer that that the reward must be hidden underneath the cup (condition D). It seems that long-tailed macaques possess at best a restricted understanding of the influence of the reward on the orientation of objects, which may not be extended to hollow objects. Thus, to this point the monkeys’ success could be based on perceptual preferences, but does not necessarily imply a general understanding of the causes of inclined boards. Therefore, in our next two experiments, we aimed to investigate in more detail what the subjects understand about the causal relationships underlying board inclinations.

## Experiment 3: inclinations of the same height

When the macaques were confronted with two inclined boards in Experiment 1, they did not consider the causally relevant information, that is, which of the boards had already been inclined before the reward was hidden and which board had been lying flat. Instead, they tended to prefer the board with the strongest inclination, which is similar to the choice strategy employed by bonobos and gorillas (Call 2007). Two possible explanations for the monkeys’ failure to detect the baited board exist. First, they may not attend to temporal information and consequently do not consider the order in which the boards became inclined. Second, they may attend to the temporal order, but a predominant bias towards choosing the board with the strongest inclination prevented them from using this information. To test this possibility, we now confronted the monkeys with two boards of identical inclination.

### Methods

#### Subjects

Seven of the eight subjects (three males) from Experiment 2 participated, while the fourth male was not motivated to enter the test compartment. In addition, another 4-year old female was tested (Table 1). This female had participated previously in the study by Schmitt et al. (2012).

#### General procedure

The general procedure was identical to Experiments 1 and 2.

#### Training

The use of the transposition task in Experiment 2 had not significantly led to a reduction inside biases. Thus, we abandoned this pre-test and used the choice task from Experiment 1 instead. Due to an experimenter error, one monkey received only 10 instead of 12 trials in his first training session. He nevertheless chose the baited cup significantly above chance (binomial test: *P* = 0.021), and we therefore advanced him to testing.

#### Testing

In this test, only the boards from Experiment 2 were presented.

We administered four different conditions:

*Hiding food*: two boards were presented with one board resting on a wooden block. The monkeys could observe how the board was put on the block to ensure that they were aware that the block caused the inclination of the board. Then the screen was put up, and a piece of banana of the same size as the block was shown to the subject and hidden behind the screen. The experimenter always began to manipulate the board on the left side and then the board on the right side. The banana was always hidden under the flat board.

*No boards*: the wooden block and an equally sized piece of banana were placed simultaneously on the platform. Then the monkeys could choose without the screen having been put up. This condition was introduced to ensure that the monkeys’ choices were not explainable by an interest for the wooden block.

*Hidden in view*: as “no boards”, but now the block and the banana were simultaneously covered with the two boards and in full view of the subjects. Then the experimenter waited 5 s before the monkeys were allowed to choose. This condition was introduced to ensure that a non-preference for the food was not due to memory failures.

*Hiding block*: as “hiding food” but the banana was first placed on the platform and covered by the board in full view of the subjects. The wooden block was then hidden under the other board behind the screen.

Initially, we planned to run only one session of the “hiding food” condition, consisting of 12 trials; after having run this session, we decided to incorporate the other conditions and therefore presented each condition en bloc within a single session. The first session comprised “hiding food” trials only and the last session comprised “hiding block” trials only. The other two conditions were presented in the second and third session; we randomised the order of these two conditions for each subject. To avoid satiating the subjects by providing them with few large pieces of reward, we reduced the size of the block (and consequently the size of the reward) to 1.5 cm, which is slightly smaller than the block presented in Experiment 1.

Due to an experimenter error in 19 of the 24 trials of two subjects in the “no boards” condition, the subjects could make their choice without having to wait 5 s. As the performance of the subjects did not differ from the other subjects, we included them in the analysis. Because of technical problems, one trial in the “no boards” condition had to be scored live.

#### Analysis

Not all data were normally distributed; we consequently used non-parametric statistics when applicable. A Friedman test with the Student–Newman–Keuls (SNK) post hoc procedure was applied to compare performances between conditions, and one-sample *t* tests or Wilcoxon tests were used to assess if group performances within each condition deviated from chance. Binomial tests were used to analyse individual performances.

### Results

#### Training

Seven subjects reached the training criterion within the first session. Only one of the females that had already participated in the previous experiments required a second session.

#### Test

**Fig. 4:**
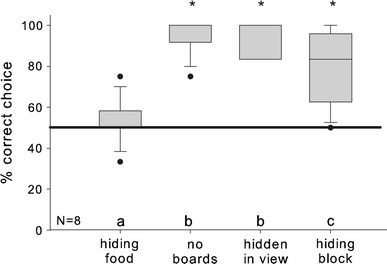
Performance of the monkeys in Experiment 3. *Boxplots* show median, 25th and 75th quartile, *whiskers* show 10th and 90th percentiles, *dots* represent outliers. The *solid horizontal line* represents the 50 % chance level. *Boxes* marked with *small letters* above the *x* axis differ significantly from each other, based on a Friedman test with post hoc SNK-tests. *Asterisks* show significant deviation from chance according to a one-sample *t* test or Wilcoxon test

We found significant performance differences between the conditions (Friedman: *N* = 8, *χ*² = 17.789, *df* = 3, *P* < 0.001). Post hoc comparisons revealed that the monkeys were equally successful in the “no boards” and in the “hidden in view” conditions (SNK: *P* > 0.05) and were more successful in these two than in the other two conditions (SNK: all comparisons *P*s < 0.05). Furthermore, they were more successful when the block was hidden behind the screen than when the food was hidden (“hiding block” vs. “hiding food”, SNK: *P* < 0.05; Fig. 4).

As further tests we compared the performance within each condition against the chance level of 50 %: the monkeys clearly preferred the banana in all but the “food hidden” condition (“no boards”: Wilcoxon: *T* ^+^ = 0, *df* = 7, *P* = 0.008; “hidden in view”: Wilcoxon: *T* ^+^ = 0, *df* = 7, *P* = 0.008; “hiding block”: paired *t* test: *t* = 4.249, *df* = 7, *P* = 0.004; “hiding food”: paired *t* test: *t* = 0.753, *df* = 7, *P* = 0.476).

On an individual level, all monkeys significantly preferred the banana in the “hidden in view” condition and seven of eight subjects did so in the “no boards” condition (binomial tests: all *P*s ≤ 0.039). In contrast, only four of the eight subjects showed a significant preference for the banana in the “hiding block” condition and none did so in the “hiding food” condition.

Four monkeys showed significant side biases in the “hiding food” condition (binomial tests: *P*s ≤ 0.039); three monkeys revealed side biases in the “hiding block” condition (binomial tests: *P*s ≤ 0.039), but no side biases occurred in the “no boards” and the “hidden in view” conditions (binomial test: all *P*s ≥ 0.146).

### Discussion

The results of this experiment suggest that the monkeys’ failure to distinguish between the two inclined boards in Experiment 1 was at least not solely due to a preference for the board with the strongest inclination. Furthermore, motivational and/or memory effects also cannot account for these results. Instead, it seems as if the macaques had difficulties to incorporate temporal information in their reasoning, that is, to consider (1) that only one board had changed its inclination after the hiding of the food, and (2) which of the two boards had changed. At first sight, however, this interpretation seems to be weakened by the better performance when the block rather than the food was hidden behind the screen (condition “hiding block”). But a successful solution of this condition does not require a comparison of the two boards and the consideration of temporal information. Instead, the subjects could have attended to the location of the banana as soon as it had been positioned on the board, ignoring the second board. Indeed, we observed that the monkeys regularly pointed at the baited board as soon as the banana had been positioned there and continued to do so until the end of the trial. Nevertheless, only four of the eight monkeys were consistently successful in this condition, which further supports the hypothesis that the monkeys had difficulties attending to the temporal sequence of the events.

## Experiment 4: the wedge task

The results of Experiment 2 revealed that the monkeys were able to use the presence of an inclination to detect the location of the reward. The question that remains is whether this is based on an understanding for the reward causing the inclination or on a preference for objects with an inclined surface (Call 2007). To test this, we modified the wedge task conducted by Call (2007) in which the subjects had to make a choice between a flat board and a solid wedge.

### Methods

#### Subjects

Seven of the eight subjects (two males) from Experiment 3 participated; the third male had to be removed from the group for husbandry reasons (Table 1).

#### General procedure

The general procedure was identical to the ones in the previous experiments. The experiment was conducted directly after Experiment 3 without previous training sessions.

#### Testing

As objects we used a solid wedge (14 × 11.5 × 2 cm) and a flat cardboard (13.5 × 11.5 cm); both were covered with blue tape. On each trial, the wedge and the board were positioned simultaneously on the platform, with the sloped side of the wedge facing the subjects. Thereby the wedge closely resembled an inclined board from the previous experiments. At the start of the session, the experimenter repeatedly showed both objects to the subjects from various angles to ensure that they were aware of the difference between the two objects. No food was visible on the platform during the entire trial.

In the original task, a food reward was hidden in a hole underneath the wedge (Call 2007). However, our subjects had no experience with such a design. We were interested in whether the monkeys had an intrinsic preference for the wedge and did not want to prime them to select it by rewarding the choice of the wedge only. Therefore, upon the presentation of both objects, the platform was pushed forward and the subjects could make their choice. Irrespective of the object they chose, the experimenter handed them a piece of banana after each trial. We ran a single session with 12 trials.

### Results and discussion

The monkeys chose the wedge on 59.5 ± 15.5 % of the trials (*x* ± SD; range 50–91.7 %), which did not deviate significantly from chance (Wilcoxon: *T* ^+^ = 0, *df* = 6, *P* = 0.125). Only one of the subjects had a clear preference for the wedge (binomial test: *P* = 0.006; all others: *P*s ≥ 0.774). Even though a statistical analysis is not possible because of the small sample size, it is noteworthy that only four of the seven subjects chose the wedge on the very first trial. Five subjects showed a significant side bias (binomial tests: *P*s ≤ 0.039). Taken together, these results show that the long-tailed macaques did not have an intrinsic preference for objects with an inclined surface. In turn, this finding suggests that the monkeys preferred the inclined boards in the previous experiments because they understood that the inclinations had been caused by an object underneath the board.

## General discussion

The present study provides evidence that long-tailed macaques seem to possess some understanding of causality, as they were capable of using the inclination of a solid cardboard to diagnose the location of hidden food; in addition, they distinguished between a solid wedge and an inclined board, which argues against a mere preference for objects with an inclined surface (Call 2007). However, the monkeys’ failure in the more complicated settings also suggests that they did not always appropriately use their causal knowledge for diagnostic reasoning. The monkeys neither succeeded when two boards were inclined so that they needed to exclude one of them based on its inclination prior to the hiding, nor when they had to use the absence of an inclination of a board to infer that the reward must have been hidden underneath a different object.

Consequently, long-tailed macaques seem to possess at least some knowledge about the influence of solid objects such as the reward on flat objects like boards. Moreover, the lack of preference for the wedge in Experiment 4 suggests that the monkeys may have preferred the inclined board in the other experiments because only this object, but not the wedge, was a possible hiding place. Importantly, causal understanding and learning are often regarded as opposing mechanisms. However, this does not need to be the case. The observation of a reward underneath the inclined board may have highlighted the cause of the inclination, facilitating the rapid acquisition of an, albeit restricted, folk-physical understanding for object–object relations. This would also be in line with the findings of a recent study on chimpanzees, showing that these apes learn a discrimination based on causal features very quickly, whereas they did not learn an arbitrary discrimination within thrice as many trials (Hanus and Call 2011).

However, it is obvious that long-tailed macaques’ abilities are restricted, as they failed to detect the reward underneath the cup. Is this deficit attributable to a failure of understanding of the presented causal relations or a partial failure of reasoning diagnostically? A lack of causal understanding would imply that the monkeys were not aware that the reward could have been hidden underneath the cup and thus may not understand “hollowness”. It is true that monkeys have been reported repeatedly to have problems with invisible displacements in object permanence tasks, that is, they often fail to track objects that had been hidden repeatedly under a series of objects (e.g., de Blois and Novak 1994; de Blois et al. 1998; Mathieu et al. 1976). However, this finding is not universal (e.g., Neiworth et al. 2003), and it is unclear how to interpret failures in invisible displacement tasks (reviewed by Gomez 2005). Furthermore, our subjects succeeded in invisible displacement tasks, even though they committed a relatively large number of errors (Schmitt et al. 2012). In addition, our monkeys had ample experience with food hidden underneath various types of cups or bowls in the course of this project as well as other investigations (e.g., Schmitt and Fischer 2009, 2011; Schmitt et al. 2012) and also mastered the transposition task used during training for Experiment 2. To acquire this competency, they required a considerable amount of training. This finding is in concordance with a study by Amici et al. (2010), who also found that long-tailed macaques master single transpositions, even though they are error-prone. Taken together, it seems safe to assume that our subjects knew that food can be hidden underneath a cup and were generally able to track the hiding of a reward. Thus, a more likely possibility is that the long-tailed macaques failed, at least in this specific context, to integrate their knowledge about object–object interactions into a correct diagnostic inference. This failure in complex situations need not necessarily imply that they do not reason diagnostically in less complex contexts. The work by Baillargeon and co-workers showed that human infants’ physical knowledge develops gradually (e.g., Baillargeon 1995, 2004) so that the cognitive skills of monkeys may be similarly restricted to certain domains or contexts. An example of such a restriction in a different task are capuchin monkeys who do not demonstrate exclusion abilities across all contexts, but only in restricted cases (Heimbauer et al. 2012; Paukner et al. 2006, 2009; Sabbatini and Visalberghi 2008).

Our task involved two steps of inference, and there is evidence that several species successfully solve one step, but fail with two-step inferences. For instance, in the most commonly used reasoning paradigms, subjects are confronted with a choice between two bowls, one of which is baited. Before the subjects make their choice, the empty bowl is lifted to inform them about the absence of the food (e.g., Call 2004; see Hampton et al. 2004; Paukner et al. 2006 for a related paradigm involving tubes instead of bowls). In this case, the solution is straightforward and only one inference is required: The animals perceive directly that the food is absent in one location and need to deduce that the food is probably in the alternative location.^1^ This task is solved by a variety of species from apes (Bräuer et al. 2006; Call 2004; Hill et al. 2011) to monkeys (Hill et al. 2011; Paukner et al. 2009; Sabbatini and Visalberghi 2008; Schmitt and Fischer 2009), dogs (*Canis familiaris*) (Erdöhegyi et al. 2007) and birds (Mikolasch et al. 2012; Schloegl et al. 2009b). Some species have also been tested in a transfer task in which they do not see but can only hear which bowl is empty: The bowls are shaken before the animals can make their choice and the shaking of the baited, but not of the empty bowl causes a rattling noise. Thus, a two-step inference is required: when only the empty bowl is shaken, they first have to infer that the lack of noise indicates the absence of the reward in this bowl, and the second inference requires them to realise that because of the absence of the food the reward must be hidden in the other bowl. Interestingly, this task has so far been solved successfully and without previous training only by apes (Call 2004; Hill et al. 2011) and Grey parrots (Schloegl et al. 2012), whereas all monkeys failed (Heimbauer et al. 2012; Paukner et al. 2009; Schmitt and Fischer 2009) or needed extensive training (Sabbatini and Visalberghi 2008). Analogous to this task, the long-tailed macaques in our experiment first needed to infer that the reward cannot be hidden underneath the flat board and second that therefore the reward must be under the cup.

The concept of diagnostic uncertainty (Fernbach et al. 2012) may help to explain the difficulties of the monkeys upon seeing two inclined boards. In the experiments of Fernbach and colleagues, human children were confronted with a box that began to play music if a wooden block was placed on top of it. In one condition, each of two dark blocks activated the machine, whereas a white block did not. After the machine had started to play music behind an occluder, the children were asked which of the blocks had caused its activation. Three- and four-year-old children master this task; when they were told that the black block they had picked did not activate the machine, they could correct themselves and select the other black block. Fernbach et al. (2012) refer to this competency as first-order diagnostic uncertainty, meaning that the subject needs to understand that more than one possible solution exists. Our experiment presented a task with lower complexity than this task, but it also provided a certain level of uncertainty: When confronted with two inclined boards, the possibility existed that under the previously inclined board an additional piece of food could have been hidden. In other words, whereas the newly inclined board was the most likely location of the reward, the previously inclined board was another possible location (see also Bräuer et al. 2006, Call 2007 and Seed et al. 2012 for a similar argument). Thus, this uncertainty may have contributed to the low performance in the conditions in which the monkeys had the choice between two inclined boards. Importantly, the same uncertainty also applies to the condition in which they had to choose between a newly inclined board and the cup (condition C of Experiment 2); indeed, the performance in this condition was worse than in the condition without any uncertainty, that is, in the condition in which one board was inclined and the other board remained flat. Nevertheless, the difference between these two conditions is not very strong, and it remains speculative whether the monkeys’ performance can be explained by sensitivity to diagnostic uncertainty.

Interestingly, the monkeys performed similarly to the Great apes (Bräuer et al. 2006; Call 2007). Apes also exhibit a preference for inclined boards. However, both apes and monkeys do not have a general preference for objects with inclined surfaces (e.g., wedges). Interestingly, the monkeys chose the inclined board even though they had not seen the process of inclining because the hiding of the reward was conducted behind a screen. This is again in concordance with the results obtained with the Great apes, but in contrast to the performance of dogs (Bräuer et al. 2006). Dogs preferred the inclined board only if they had seen it inclining (but without observing that a food reward caused the inclination). This led the authors to suggest that in contrast to apes, dogs lack an understanding of the underlying causal relations but were subject to enhancement effects instead (Bräuer et al. 2006). To our knowledge, no previous study has yet employed our test paradigm in which the subjects have to choose between a (flat or inclined) board and a cup. We can make clear and testable predictions how other species and in particular apes should perform. If we are correct with our hypothesis that the monkeys’ failure to infer the location of the reward underneath the cup is due to an inability to conduct two-step reasoning operations, the apes should master this task. This hypothesis is based on the successful performance of apes, but not monkeys, in the shaking experiment described above, which may also be based on two-step inferences (Call 2004; Heimbauer et al. 2012; Paukner et al. 2009; Schmitt and Fischer 2009; but see Seed et al. 2012 for failures of some apes in a different task).

Finally, a critic may argue that the monkeys may have responded to subtle experimenter-provided cues. This, however, cannot explain why only two subjects selected the single inclined board in the first experiment (conditions C and D). From the previous study by Schmitt et al. (2012), it is already known that monkeys prefer inclined boards and thus it is obvious that the experimenter expected the monkeys to succeed in these conditions. Similarly, we had expected that the monkeys would be more successful throughout the study, but they apparently were not. Thus, unintended cueing seems highly unlikely to explain the subjects’ performances.

In summary, our results demonstrate that long-tailed macaques seem to possess an at least restricted understanding of the causal influence objects can have on the spatial orientation of other covering objects. They fail, however, to adequately incorporate this knowledge in complex diagnostic reasoning that requires two-step inferences.

## Footnotes

1 Because of this straightforwardness, it is heavily disputed if this task provides any evidence for reasoning. Instead, it may be solved through avoidance of the empty bowl alone (e.g., Paukner et al. 2009; Schloegl et al. 2009a; Schmitt and Fischer 2009).
